# Collectanea Medica: Consisting of Anecdotes, Facts, Extracts, Illustrations, &c.

**Published:** 1821-09

**Authors:** 


					COLLECTANEA MEDIC A:
CONSISTING OF
ANECDOTES, FACTS, EXTRACTS, ILLUSTRATIONS, &C.
Relating to the History or the Art of Medicine, and the
Auxiliary Sciences.
Floriferis ut apes in saltibns omnia libant,
Omnia nos itidem depascimur aurea dicta.
Abstract of Baron Humboldt's Dissertation on Isothermal Lines,
and the Distribution of Heat over the Globe*
rpHE distribution of heat over the globe belongs to that kind of
A phenomena, of which the general circumstances have been long
known, but which were incapable of being rigorously determined or
submitted to exact calculation, till experience and observation ur-
nished data from which the theory might obtain the corrections an
the different elements which it requires. The object of this memoir is
to facilitate the collection of these data, to present results drawn trom
a great number of unpublished observations, and to group them ac-
* This highly valuable and extremely interesting Dissertation has
claims on the space of this Journal; but it occupies too much room topennu us
to give it in detail: only those points relating especially to medical scienc ? ,
therefore, be selected; but the extent of these even will render itnecessaty to
divide our abstract into portions which may be inserted in the present ana w
three subsequent Numbers.?*Ewr.
<288 Collectanea Medica.
cording to a method which has not yet been tried, though its utrii^
has been recognized for more than a century in the exposition of the
phenomena of the variation and dip of the magnetic needle. As the
discussion of individual observations will be published in a separate
?work, I shall at present limit myself to a simple sketch of the distri-
bution of heat over the globe, according to the most recent and accu-
rate data. Although we may not be able to refer the complex
phenomena to a general theory, it will be of considerable importanca
to fix the numerical relation by which a great number of scattered
observations are connected, and to reduce to empirical laws the effects
of local and disturbing causes. The study of these laws will point out
to travellers the problems to which they should direct their principal
attention; and we may entertain the hope that the theory of the dis-
tribution of heat will gain in extent and precision, in proportion as
observations shall be more multiplied, and directed to those points
which it is of most importance to illustrate.
As the phenomena of geography and of vegetables, and in general
the distribution of organized beings, depend on the knowledge of the
three co-ordinates of latitude, longitude, and altitude, I have been oc-
cupied for many years in the exact valuation of atmospherical tempe-
ratures; but I could not reduce my own observations without a
constant reference to the works of Cotte and Kirwan, the only ones
which contain a great mass of meteorological observations obtained by
instruments and methods of very unequal precision. Having inhabited
for a long time the most elevated plains of the New Continent, I
availed myself of the advantages which they present for examining the
temperature of the superincumbent strata of air, not from insulated
data, the results of a few excursions to the crater of a volcano, but
from the collections of a great number of observations made day after
day and month after month in inhabited districts. In Europe, and in
all the Old World, the highest points of which the mean temperatures
have been determined are the conTent of Peissenberg, in Bavaria, and
the hospicc of St. Gothard.* The first of these is placed at 326'4, and
the second at 6808, feet above the level of the sea. In America, a
?great number of good observations have been made at Santa Fe de
Begota and at Quito, at altitudes of 8,727 and 9*544 feet. The town
of Iluancavelica, containing 10,000 inhabitants, and possessing all the
resources of modern civilization, is situated in the Cordilleras of the
southern hemisphere, at 12,310 feet of absolute elevation; and the
mine of Santa Barbara, encircled with fine edifices, and placed a league
to the south of Huancavelica, is a place fit for making regular obser-
vations, at the height of 14,509 feet, which is double that of the hos-
pice of St. Gothard.
These examples are sufficient to prove how much our knowledge of
the higher regions of the atmosphere, and of the physical condition of
the world in general, will increase, when the cultivation of the sciences,
? The mean temperature of the air at the convent of the Great St. Bernard, the
height of which is 7,960 feet, is not determined. There are several villages in
Europe placed at more than 5000 feet of altitude; for example, St. Jacques d?
Ayas at 5,479, and Trinita Nuova, near Grasfoncy, at 5,315 feet.
6
jJaron Humboldt on Isothermal Lines. 289
so long con fined to the temperate zone, ,shall extend beyond the tropics
into those vast regions where the Spanish Americans have already de-
voted themselves with such zeal to the study of physics and astronomy.
In order to compare, with the mean heat of temperate climates, the
results which Mr. Bonpland and I obtained in the equinoctial regions
from the plains to the height of 19,292 feet, it was necessary to collect
a great number of good observations made beyond the parallels of
30? and 35?. I soon perceived how vague such a comparison was, if I
selected places under the meridian of the Cordilleras, or with a more
eastern longitude; and I therefore undertook to examine the results
contained in the most recent works. I endeavoured to find, at every
ten degrees of latitude, but under different meridians, a small number
of places whose mean temperature had been precisely ascertained, and
through these, as so many fixed points, passed my isothermal lines, or
lines of equal heat. 1 had recourse, in so far as the materials have
been made public, to those observations the results of which have been
published ; and I found, in the course of this easy, but long and mo-
notonous, labour, that there are many mean temperatures pointed out
in meteorological tables, which, like astronomical positions, have been
adopted without examination. Sometimes the results were in direct
contradiction to the most recent observations, and sometimes it was
impossible to discover from whence they were taken.
Many good observations were rejected, solely because the absolute
height of the place where they were made was unknown. This is the
?case with Asia Minor, Armenia, and Persia, and of almost all Asia;
and, while the equinoctial part alone of the New World presents al-
ready more than five hundred points, the greater number of which arc
simple villages and hamlets, determined by barometrical levelling, we
are still ignorant of theheight of Erzeroum, Bagdad, Aleppo, Teheran,
Ispahan, Delhi, and Lassa, above the level of the neighbouring seas.
Notwithstanding the intimate relation in which we have lately , stood
"With Persia and Candahar, this branch of knowledge has not made any
progress in the last fifty years. ;
We are not authorized, however, on account of the decrease of tem-
perature in the upper regions of the atmosphere, to confound the
mean temperatures of places which arc not placed on the same level.
In the Old World, good observations, which can alone be used for
establishing empirical laws, are confined to an extent between the pa-
rallels of 30 and 70 degrees of latitude, and the meridians of 30? east
longitude and 20? of west longitude. The extreme points of this re-
gion are the island of Madeira, Cairo, and the North Cape. It is a
zone which is only a thousand nautical leagues, (one-seventh of the
circumference of the globe,) from east to west, and which, containing
the basin of the Mediterranean, is the centre of the primitive civiliza-
tion of Europe. The extraordinary shape of this part of the world,
the interior seas and other circumstances, so necessary for developing
*he germ of cultivation among nations, have given to Europe a parti-
cular climate, very different from that of other regions placed under
the same latitude. . -
The temperature of the atmosphere and the magnetism of the globe,
NO. 271. 2 p
2Q0 Collectanea. Medica.
cannot, like those phenomena which depend on one cause or on a
single centre of action, be disengaged from the influence of disturbing
circumstances, by taking the averages of many observations in which
these extraneous effects are mutually destroyed. We must, however,
guard against confounding, under the name of extraneous and disturb-
ing causes, those on which the most important phenomena, such as
the distribution and the more or less rapid development of organic
life, essentially depend. Our object is to ascertain the quantity of
heat which every point of the globe annually receives, and, what is of
most importance to agriculture and the good of its inhabitants, the
distribution of this quantity of heat over the different parts of the
year, and not that which is due to the solar action alone, to its alti-
tude above the horizon, or to the duration of its influence, as measured
by the semidiurnal arcs.
Moreover, we shall prove that the method of means is unfit for as-
certaining what belongs exclusively to the sun, (inasmuch as its rays
illuminate only one point of the globe,) and what is due both to the
sun and to the influence of foreign causes.
In distinguishing, as has long been done, between the solar and the
real climate, we must not forget that the local and multiplied causes
which modify the action of the sun upon a single point of the globe,
are themselves but secondary causes, the effects of the motion which
the sun produces in the atmosphere, and which are propagated to great
distances. If we consider separately (and it will be useful to do this
in a discussion purely theoretical,) the heat produced by the sun, the
earth being supposed at rest and without an atmosphere, and the heat
due to other causes regarded as disturbing ones, we shall find that this
latter part of the total effect is riot entirely foreign to the sun. The
influence of small causes will scarcely disappear by taking the mean
result of a great number of observations; for this influence is not li-
mited to a single region. By the mobility of the aerial ocean, it i
propagated from one continent to another. Every where in the re-
gions near the polar circles, the rigors of the winters are diminished
by the admixture of the columns of warm air, which, rising above the
torrid zone, are carried towards the poles: every where in the tempe-
rate zone, the frequent west winds modify the climate, by transporting
the temperature of one latitude to another.* When we reflect, be-
sides, on the extent of seas, on the form and prolongation of continents,
either in the two hemispheres, or to the cast and west of the meridians
of Canton and of California, we shall perceive that, even if the number
of observations on the mean temperature were infinite, the compensa-
tion would not take place.
It is, then, from the theory alone that we must expect to determine
the distribution of heat over the globe, in so far as it depends on the
immediate and instantaneous action of the sun. It does not indicate
the degrees of temperature expressed by the dilatation of the mercury
in a thermometer, but the ratios between the mean annual heat at the
equator, at the parallel of 45?, and under the polar circle; and it
* Raymond, Mcmoire sur la For mule Baromet. p. 108 aud 115.
Baron Humboldt on Isothermal Lines. 291
determines the ratios between the solstitial and equinoctial heats in
different zones. By comparing (he results of calculation, not with the
mean temperature drawn from observations made under different lon-
gitudes, but with that of a single point of the earth's surface, we shall
set out with that which is due to the immediate action of the sun and
to the whole of the other influences, whether they are solar or local,
or propagated to great distances. This comparison of theory with ex-
perience will present a great number of interesting relations.
In the year 1693, previous to the use of comparable thermometers,
and to precise ideas of the mean temperature of a place, Halley
laid the first foundations of a theory of the heating action of the sun
under different latitudes.* lie proved that these actions might com-
pensate for the effect of the obliquity of the rays. The ratios which
he points out do not express the mean heat of the seasons, but the heat
of a summer-day at the equator and under the polar circle, which he
finds to be as 1-834 to 2'310.
In detailing the actual state of our knowledge on the distribution of
heat, I have shown how dangerous it is to confound the results of
observation with theoretical deductions. The heat of any point of the
globe depends on the obliquity of the sun's rays and the continuance
of their action, on the height of the placc, on the internal heat and
radiation of the earth in the middle of a medium of variable tempera-
ture; and, in short, upon all those causes which are themselves the
effects of the rotation of the earth and the inequal arrangement of
continents and seas. IJeforc laying the foundation of a system, we
must group the facts, fix the numerical ratios, and, as I have already
pointed out, submit the phenomena of heat, as Halley did those of
terrestrial magnetism, to empirical laws. In following this method, I
have first considered whether the method employed by meteorologists
for deducing the mean temperature of the year, the month, and the
day, is subject to sensible errors. Assured of the accuracy of the nu-
merical averages, I have traced upon a map the isothermal lines,
analogous to the magnetic lines of dip and variation. I have consi-
dered them at the surface of the earth in a horizontal plane, and on
the declivity of mountains in a vertical plane. I have examined the
increase of temperature from the pole to the equator, which is inequal
under different meridians; the distribution of the same quantity of
heat over the different seasons, in the same isothermal parallel, and
nuder different latitudes; the curve of perpetual snows, which is not
a line of equal heat; the temperature of the interior of the earth,
?which is a little greater towards the north, and in high mountains, than
the mean temperature of the atmosphere under the same parallel; and,
lastly, the distribution of heat in the ocean, and the position of those
bands, which may be called bands of the warmest waters. As the
limits of this extract will not permit me to enter, in a detailed manner,
upon these different discussions, I shall confine myself solely to the
principal results.
It was formerly the custom to take the maximum and minimum of
Phil. Trans. 1693, p. 878.
2 p 2
292 Collectanea Medica.
temperature observed in the coarse of a year, and to consider half tire-
sum as the mean temperature of the whole year.
In order to diminish the errors of the method of annual extremes, it
was perceived, though very late, that it was necessary to subdivide the
curve which expresses the variation of temperature. Twenty-four
extremes divided among twelve months of the year, give an annual'
mean more exact than the. two extremes of all the observations. The
ordinates do not increase uniformly and uninterruptedly up to the
maximum of the year, and there are partial inflexions sufficiently re-
gular. The more we subdivide, and the more we know the terms in
the series, the more will these terms approximate, and the less error
will there be in the supposition of an arithmetrical progression, and in
that of the equidistance of the different maxima and minima of tempe-
rature. These considerations enable us to appreciate the three methods
according to which observations are at present made. 1. Observations
are made three times a-day, at sunrise and sunset, and at two o'clock
in the afternoon. This was done at Geneva, during the three years
1796, 1797, and 1798. In the observations, the hour of mid-day was
preferred to that of sunset. 2. Observations are made tivice every
day, at the two periods which are supposed to give the maximum and
the minimum,?namely, at sunrise and at two o'clock in the afternoon.
3. Observations are made once a-day, at an hour which, in different
seasons, has been found to represent the mean temperature of the day.
It is thus that Mr. Raymond, by a judicious induction, has proved
that the height of the barometer at mid-day gives, in our climates, the
mean atmospherical pressure, corrected for the diurnal variation.
In calculating* a great number of observations made between the
parallels of 46? and 48?, I have found that a single observation at
sunset gives a mean temperature, which differs only some tenths of a
degree from that which is deduced from observations made at sunrise
and at two o'clock. The deviations of different months do not exceed
1*8, and they are very regularly positive or negative, according to
the order of the seasons. Mr. Aragof has examined for seven years
the observations of noon. They givo for Paris 5*4 more than the
mean temperature of the whole year. Upon high mountains in the
temperate zone, the difference is scarcely 1*8. $ By the application
of coefficients, variable according to the latitude and the elevation, we
may deduce the true mean temperatures from observations made at
any particular period of the day, nearly in the same manner as we can
ascertain the latitude of a place from altitudes of the sun, taken out of
the meridian.
If we do not stop at two observations of the maximum and mini-
mum, but add a third observation, we commit an error more or less
serious, if we divide simply by three the sum of the observations,
without attending to the duration of the partial temperatures and to
* De la Formule Barom. p. 213.
t The mean of the observations at noon at Paris, was 56*84; at Clermont in
Auvergne, (elevation, 1348 feet,) 56'SO; at Strasburg, (elevation, 453
55'22.?jBulletin de la Soc. Pldlom. 1814, Oct. p. 55-
j At the hospice of St. Gothard.?Ephem, Soc. Pal, 1785, p. 47.
Baron Humboldt on Isothermal Lines.' 2Q3
the place which the third observation occupies between the last terms
of the series.* Experience proves that the mean temperatures of the
year, obtained by two or three observations, do not differ sensibly, if
the intermediate observation is sufficiently distant (four or fire hours)
from the observation of the maximum and minimum. Whenever,
therefore, we do not take into account the duration of the intermedi-
ate temperatures, we should prefer the two observations of the extreme
temperature, which is the method most generally adopted. We shall
content ourselves with pointing out the errors to which it is liable. In
our climates, the two extreme terms do not divide the series of twenty-
fours into two equal parts. The maximum is an epoch nearly fixed:
the rising of the sun retards or hastens it three hours. As we ought
to take into account the duration of the partial temperature, in order
to find the quantity of heat divided between the night and the day, we
must couple the maximum of one day with the minimum of the day
following, and not be content with taking half the sum of all the
maxima and minima of a month. In the ordinary method, we deter-
mine only the mean temperature of the part of the day comprehended
between the rising of the sun and two o'clock in the afternoon; and
we take it for granted that the mean temperature is the samef from
two o'clock to sunrise next day. This double error, of want of equi-
distance and of the coupling of observations, does not in general pro-
duce errors of more than some tenths of degrees, sometimes in exccss,
and sometimes in defect, since the warm and cold days are mixed. J
All the calculated results will err in defect, if the 365 ordinates
through which the curve of the year passes do not express an arithme-
tical progression, and if the partial irregularities do not sensibly com-
pensate one another. It is only on this supposition that we can judge
by the extreme terms of the series, of the sum of the terms; that is, of
the partial temperatures. It is very obvious, that near the maximum
the increase ought to be more slow than in other points of the curve
and that this increase in the temperature of the air ought to depend on
the sine of the sun's altitude, and on the emission of the radiant heat
of the globe.
* Example.?On the 13 th June, at 4h in the morning, 46'4 ; at 2h in the after-
noon, 55-4; and at llh in the evening, 50?, (erroneously, 46*4, or 8? centig. in
the original.) In calculating by the duration, we have
50*9, the mean for 10h of interval, ? 509-0
52-7 9 ? 474-3
48*2 5 = 241*0
The true mean of which is 51*0. The mean of the three observations, as com-
monly taken, is 50*6. If we stop at the two extreme temperatures, we shall have
for their half sum 50*45.
t Example.?At sunrise at 6h, 50?; at 2 o'clock in the afternoon, 62'6. At
sunset, 51*8; at 2h, 66*2; at sunrise, 50?. The true means will be, for the first
24 hours, 56*9; and for the second, 59'0; for we shall have
For 8h, \ (50-0 + 66*2) X ? = 450*4 for 8h r= 472*0
16h, 1(51*8 + 62*6) X 16 rr 915*2 for 16h ? 929*6.
? The method commonly employed gives | (50? + 62*6) ? 56*3, and i(66'2 + 51 8)
= 58-1. The errors being ? 0*6 and + 0-9, sometimes positive and sometimes
t The error disappears when days of equal temperature succeed one another.'
It amounts to 1*8, if the mean temperatures of two successive days diner from
to 9?, which however very rarely happens.
&g4 Collectanea Medica.
II appeared to me very important to establish, by observations made
at every hour, at different periods of the year, and under different la-
titudes, the degree of confidence that can be placed in those results
which are called mean temperatures. I have selected from the registers
of the Royal Observatory at Paris clear and calm days, which offered
at least tea or twelve observations. Under the equator, I have spent
?whole days in determining the horary increments and decrements of
temperature, in marking the thermometer both in the shade and in the
sun, and also the progress of evaporation and humidity; and, in order
to avoid calculation, I measured with a quadrant the altitude of the
sun at each observation. I chose days and nights completely calm,
and when the heavens were entirely free from clouds, because the
mass of vesicular vapours interrupts the radiation from the earth.
The result of these experiments has been very satisfactory, and proves
what had already been deduced from the coincidence between the tem-
perature of the earth and the mean of daily observations, and from
the regular progress of the mean temperatures of months in different
years, that the effects of small disturbing causes may be compensated
by a great number of observations.* I have obtained analogous results
by taking, for several months, the mean of g o'clock in the morning,
of sunrise and midnight. I have computed the temperatures by the
distance of the maximum expressed in time, and on the supposition of
an arithmetical progression. I have found that, under the Torrid
Zone, the morning curve, from sunrise to the maximum, differs very
regularly from the evening curve. In the morning, the true mean
temperature, such as we find by taking the duration into account, is
a little greater than half the sum of the extremes. + Jn the evening,
* On the 3d and 4th September, 1811, lat. 48? 50'.
True mean temperature,
Sum of the temperatures taking into account the Half sum of the tico ex-
during 24 hours. duration. irane temperatures.
625-71 Falir. 57*92 Fahr. 58'28 Fahr.
. 672*49 59*90 61*88
834*67 66*74 65* 1*2
834-67 66*74 68*00
835 37 66*74 63-50
63-61 Mean. 63*35 Mean.
The three last days show an equality of temperature which is very surprising, aius
which does not appear but iu the true means.
t Example.?Latitude 10? 25'.
Calculation of a Supposition oj
true mean by an arithmetical
the duration. progression.
Before the maximum, nth September 1799,. 70-52 Falir. 69 44 Fahr.
, 14th 69-26 68-00
18 th 71-24 70*34
After the maximum, 18th August, 68*72 69-80
20th 70-16 71-24
27th 68-72 69-26
Before tli^ maximum, 17th August, 69*26 68*00
After the maximum, 17th August, 65*48 66-02
Total effect, 17th August, 67-37 67-01
Baron Humboldt on Isothermal Lines. 295
the error is in a contrary direction, and the series of temperatures ap-
proaches more to a progression by quotients. The differences do not
in general exceed half a degree, and calculation proves that the com-
pensation is regular. It would be curious to examine the effect which
the radiation of the earth has on these horary effects, as the changes of
temperature at the surface do not follow the geometrical progression,
in so far as they take place in a medium of uniform temperature.
In order to avoid the use of an arbitrary measure, astronomers ex-
press the diameters of the planets by taking that of the earth for unity.
In like manner, I express the mean temperatures, not in parts of the
equatorial heat, but by the arithmetical ratios which subsist between
this heat and that of the other parallels. This method frees us from
the want of uniformity, which arises from the use of different thermo-
meters. Instead of saying, that in Europe, under the parallel of 45?,
the mean temperature is 13*4 Centigrade, or 56*12 of Fahrenheit, we
say that it is = 1'0?,487, and in lat. 55? = 1*0?,29. These arithme-
tical ratios inform us of what is most interesting in the theory of the
distribution of heat, that, in thermometers whose zero is the point of
melting ice, the mean temperatures under the latitude of 45? and 55?
are, in our regions, the half and the third nearly of the equatorial
temperature, which I suppose to be 81*5.
Having discussed the method of taking averages, and of reducing
temperatures to general expressions, we shall now proceed to trace
the course of the isothermal lines on the surface of the globe, and at
the level of the sea. From a slight attention to the difference of cli-
mates, it has been remarked, more than a century ago, that the tem-
peratures are not the same under the same parallels; and that, in
advancing 70? to the east or the west, the heat of the atmosphere suf-
fers a sensible diminution. In pursuance of our method, we shall
reduce these phenomena to numerical results, and show that places si.
tuated under the same latitudes do not differ, in America and Europe,
by the same number of degrees of temperature, as has been vaguely
stated. This assertion would make us suppose that the isothermal
lines are parallel in the temperate zone.
Natch.cz
Funchal
I. Parallels of Georgia, of the State of\ Orotava
Mississippi, of Lower Egypt, and< Rome ??
Madeira. } Algiers
Difference
? Williamsburg
Bourdeaux**
IT* Parallels of Virginia, Kentucky, 1 jRorae^-'-^*
Spain, and the South of Greece. \ Algiers ???<
Difference
Lat.
51? 28'
32 37
28 25
41 53
S6 18
7 0 | 4
38 8
44 50
43 36
41 53
36 48
7 0
? Collectanea Medica.
III. Parallels of Pennsylvania, Jersey,j
Connecticut, Latium, and Romelia.
'Philadelphia
New-York ?
St. Malo
Nantes ? ???
Naples ? ??
Difference ? ???
Ipswich
Cambridge (Amer.)
"Vienna
Manheim
Toulon
Rome
Difference ?...
f Quelec ?
i Upsal ?
IV. Parallels of Canada, Nova Scotia, J Padua*
France, and the South of Germany. Paris ?
Difference ????
Nains ?....
Olcalc ????-?
Umea
V. Parallels of Labrador, the South of J Enontekies.
Sweden, and Courland. j Edinburgh ?
^ Stockholm ?
Difference ????11 0 if l
Lat.
39? 56'
40 40
48 39
47 13
40 50
7 0
42 38
42 25
48 13
49 29
43 7
41 53
6 30
46 47
59 51
45 24
48 50
13 0
57 0
57 20
63 50
68 30
55 58
59 20
This table* indicates the difference of climates, expressed by that of
the mean temperature, and by the number of degrees in latitude
?which it is necessary to go northward in Europe, in order to find the
same quantity of annual heat as in America. As a place could not be
found in the Old World whose mean temperature was 48?, the same
as that of Williamsburg, I have supplied it with an interpolation be-
tween the latitudes of two points whose mean temperatures arc 56'5
and 59*4. By an analogous method, and by employing only good
observations, I have found that
]. The isothermal line of 32? (0? centig.) passes between Uleo and
Enontekies in Lapland, (lat. 66? to 68? ; East long, from London
19? to 22?,) and Table Bay in Labrador, (lat. 54? 0'; W. long. 58?.)
2. The isothermal line of 41? (5? centig.) passes by near Stockholm,
(lat. 60?, East long. 18?,) and the Bay of St. George in Newfound-
land, (lat. 48?, and long. 59?.)
3. The isothermal line of 50? (10? centig.) passes by Belgium,
(lat. 51 East long. 2?,) and near Boston, (lat. 42? 30', West long.
70? 59.) _ '
* See my Prolegomena de Distributione Geographica Pla.ntarv.rn, secundum Cctli
temperiem et altitudinem montium, p. 68.
6 -
Baron Humboldt on Isothermal Lines, 297
+. The isothermal line of 59? (15? centig.) passes between Rome
&nd Florence, (lat. 43? 0', East long. 11? 40',) and near Raleigh in
North Carolina, (lat. 36? 0', and West long. 76? 30'.)
The direction of these lines of equal heat gives, for the two systems
of temperature which we know by precise observations,?-viz. part of
the middle and west of Europe, and that of the coast of America, the
following differences:
Latitude. Mean Temp, of the West
qf the Old World.
30 70-52
40 63-14.
50 50-90
60 40-64
Mean Temp, of the East
of the New World.
66-92
51-50
37-94
23-72
Difference.
3-60
8-64
12-96
16-92
If we call (he mean equatorial temperature 1, we shall have the half
of this temperature in the Old World at 45?, and in the east of the
New World at 39? of lat.
The mean temperatures decrease
Latitude.
0??20?"
20 ?30
30 ?40 I In the Old J 7*2 I In the New
40 ?50 f World, \ n-6 f World,
50 ?60
0 ?60
In both continents, the zone in which the mean temperature decreases
ihost rapidly is comprehended between the parallels of 40? and 45?.
Observation here presents a result entirely conformable to theory, for
the variation of the square of the cosine, which expresses the law of
the temperature, is a maximum towards 45? of latitude.
We have traced the direction of the isothermal lines from Europe
to the Atlantic provinces of the New World. We have seen them ap-
proach one another from parallelism towards the south, and converge
towards the north, particularly between the thermometric curves of
41? and 50?: we shall now endeavour to pursue them to the west.
North America presents two chains of mountains, extending from N.E.
to S.W. and from N.W. to S.E. forming almost equal apgleswith the
meridian, and nearly parallel to the coasts which are opposite to
Europe and Asia,?viz. the chain of the Alleghanys and the Rocky
Mountains, which divide the waters of the Missouri and the Columbia.
Between these chains stretch the vast basin of the Mississippi, the
plains of Lousiana and of the Tenesse, and the states of Ohio, the
centre of a new civilization. It is generally believed in America that
the climate is more mild to the west of the Alleghany mountains, than
under the same parallels in the Atlantic states. Mr. Jefferson has
estimated the difference at 3? of latitude; and the gleditsia mono,
sperma, the catulpa, and the aristolochia syyho, and other vegetable
productions, are found so many degrees farther to the north, in the
basin of the Ohio, than on the coast of the Atlantic.*1 Mr. Volney has
* See my Essai sur la Geographic de.i Pinnies, p. 154.
No. 271. 2 o.
298 Collectanea Medical
endeavoured to explain these phenomena by the frequency of the
south-west winds, which drive back the warm air of the Gulf of
Mexico towards these regions. A series of good observations, made,
for seven years, by Colonel Mansfield at Cincinnati, on the banks of
the Ohio, and recently published by Mr. Drake, in an excellent trea-
tise on American Meteorology,* has removed the doubts which ob-
scured this point. The thermometrical means prove that the isothermal
lines do not rise again in the regions of the west. The quantity of
heat which each point of the globe receives under the same parallels is
nearly equal on the east and west of the Alleghany range, the winters
being only a little milder to the west, and the summers a little warmer.f
The migrations of vegetables towards the north are favoured, in the
basin of the Mississippi, by the form and the direction of the valley
which opens from the north to the south. In the Atlantic provinces,
on the contrary, the valleys are transverse, and oppose great obstacles
to the passage of plants from one valley to another.
If the isothermal lines remain parallel, or nearly so, to the equator,
from the Atlantic shores of the New World to the east of the Mississippi
and the Missouri, it cannot be doubted that they rise again beyond
the Rocky Mountains, on the opposite coast of Asia, between 35? and
55? of latitude. Through 122? 40' of west long, the isothermal line
of 50? Fahr. appears to pass almost as in the Atlantic part of the Old
World, at 50? of lat. The western coasts of the two worlds resemble
one another to a certain point.j But these returns of the isothermal
lines do not extend beyond 60?. The curve of 32* Fahr. is already
found to the south of the Slave Lake, and it comes still farther south
in approaching Lakes Superior and Ontario.
In advancing from Europe towards the east, the isothermal lines
again descend,? the number of fixed points being few. We can only
? Natural and, Statistical View or Picture of Cincinnati and the Miami Country.
1 vol. 8vo. Cincinnati.
t The following comparison of the mean temperatnrea has been deduced with
great care.
Cincinnati.
Lat. 36? 6', West long. 84? 24'.
Winter, 32'9 Fahr,
Spring, 54-1
Summer, 72*9
Autumn, 54*9
Mean, 53-7
Philadelphia.
Lat. 39? 56', West long. 75? 16'.
Winter, 32*2 Fahr.
Spring, 51*4
Summer, 73-9
Autumn, 56*5
Mean, 53-5
I have taken for Philadelphia the means between the observations of Coxe and
Rush. I have also referred, for correction, to the observations made by Mr.
Le?a?x at Spring-Mill, upon the Schuylkill, to the north of Philadelphia. As
Cincinnati is 512 feet above the level of tha sea, its mean temp, is 1*4 too low.
j On account of the influence of west and south-west winds. See Dalton's
Meteor. Obseiv. p. 125.
$ In comparing places from the west to the east, and nearly under the same
parallel, we find, Mean
West. TmI. Temp.
St. IVIalo, 48*39 515
Amsterdam, 52*21 53*4
Naples, 40*50 63"3
Copenhagen, 55-41 457
Upsal, 59 52 41*9
East. Lat.
Vienna, 48*13
Warsaw, 52*14
Pekin, 39*54
Moscow, 55*46
Felersburgli, 59*56
Baron Humboldt on isothermal Lines. 290
employ those which are made in places whose known deration allows
us to reduce the mean temperatures to the level of the sea. The few
good materials which we possess have enabled us to trace the curves of
32? and 55*4. We know even the nodes of the latter curve round the
whole globe. It passes to the N. of Bourdeaux, (lat. 45*46, W.long.
0*37,) near Pekin, (lat. 39*54, E. long. 116*275) and Cape Foul,
weather to the S. of the embouchure of the Colombia, (lat. 44*40, W.
long. 104?.) Its nodes are distant at least 162? of longitude. We have
here pointed out only the empirical laws, under which are ranged the
general phenomena and the variations of the temperature which em-
brace at once a vast extent of the globe. There arq partial inflexions
of the isothermal lines, which form, so to speak, particular systems
modified by small local causes; such as the strange inflexion of the
thermometric curves on the shores of the Mediterranean} between
Marseilles, Genoa, Lucca, and Rome,* and those which determine the
difference between the climate of the western coast and the interior of
France. These last depend much less on the quantity of heat received
by a part of the globe during the whole year, than upon the unequal
distribution of heat between winter and summer. It will one day be
useful to have, upon particular charts, the partial inflexions of the
isothermal lines, which are analogous to the lines of soundings or of
equal declivity. The employmentof graphical representations will throw
much light upon phenomena which are deeply interesting to agricultu-
rists. If, in place of geographical charts, we possessed only tables
containing the co-ordinates of latitude, longitude, and altitude, a
great number of curious facts relative to the configuration and the
superficial inequalities of continents would have remained for ever un-
known.
W e have already found that, towards the north, the isothermal lines
are neither parallel to the equator nor to one another; and it is on
account of the want of parallelism that we have, in order to simplify
such complicated phenomena, traced round the whole globe the curves
of equal heat. The position of the line of 32? acts like the magnetic
equator, whose inflexions in the South Sea modify the inclinations at
great distances. We may even believe that, in the distribution of cli-
mates, the line of 32? determines the position of the curve of greatest
heat, which is as it were the isothermal equator ; and that, in America
and Asia, through 78? of west and 102? of east longitude, the torrid
zone commences more to the south of the tropic of Cancer, or that it
there presents temperatures of less intensity. An attentive examina-
tion of the phenomena proves that this is not the case. Whenever wc
approach the torrid zone below the parallel of 30?, the isothermal
lines become more and more parallel to one another and to the earth s
? ? 7_ t . qft4 feet. The ab-
Tiie elevation of Pekin is inconsiderable; tbatofft but the city i?
eolute temperature of Madrid, to the west of Naples, is o? ,
elevated 197S feet above the level of the sea.
Mean
Lat, Temp.
Bologua, 44*29 66-3
Genoa, 44'23 60*6
Mean
Lat. Temp.
Marseilles, 43*17 .58*8
Rome, 41*53 60*4
oOO Collectanea Medico,.
equator. The great colds of Canada and Siberia do not extend tlieif
action to the equatorial plains. If we have long regarded the Old
World as warmer between the tropics than the New World, it is, 1st,
because, till 176'0, travellers used thermometers of spirit of wine, co-
loured, and affected by light; 2d, because they observed it cither
under the reflection of a wall or too near the ground, and when the
atmosphere was filled with sand; and 3d, because, in place of calcu-
lating the true mean, they used only the thermometrie maximum and
minimum. Good observations give,
Old World. Lat. Mean Temp.
Senegambia, 15-0 79-07 *
Madras, 13* 5 80*42
Batavia, 6' 12 80 42
Manilla, 14-36 78-03
New World. Lai. Mean Temp.
Cumana, 10*27 81*86
Antilles, 17* 0 81.05
Vera Cruz, 19-11 78-08
Havannah, 23*10 78*08
The mean temperature of the equator cannot be fixed beyond 8l-??.
Kirwan values it at 84?; but only two places of the earth were
known, viz. Chandernagor and Pondicherry, to which old travellers
attributed annual temperatures above 8lf?. At Chandernagor, in
latitude 2 r6", the mean temperature, according to Colte, is 01 *9; but
the Jesuit Boudier marked only the days when the thermometer was
above 98-6 and below 57*2: and at Pondicherry, in latitude 11*53,
the mean temperature, according to Cotte, is 85*3, and according to>
Kirwan, 88?; but M. de Cossigny observed with a spirit.of-wine
thermometer.
The distribution of heat over different parts of the year differs, not
only according to the decrease of the mean annual temperatures, but
also in the same isothermal line. It is this unequal division of the heat
which characterizes the two systems of climate of Europe and Atlantic
America. Under the torrid zone, a small number of months are
warmer in the Old World than in the New. At Madras, for example
according to Dr. Roxburgh, the mean temperature of June is 89*4;
at ^busheer, 93/2; but at Cumana I have found it only 84*6'.
With respect to the temperate zone, it has long been known that,
from the parallel of the Canary Isles to the Polar Circle, the severity
of the winter augments in a progression much more rapid than the
summers diminish in heat. It is also known that the climate of the
islands and the coasts differs from that of the interior of continents,
the former being characterized by mild winters and less temperate
summers. But it is the heat of summer particularly which affects the
formation of the amylaceous and saccharine matter in fruits, and the
choice of the plants that ought to be cultivated. As the principal ob-
ject of this memoir is to fix, after good observations, the numerical
relations between the unequal quantities of heat distributed over the
globe, we shall now compare the mean temperatures of three months
of winter and summer under different latitudes, and show how the in-
flections of the isothermal lines modify these relations. In following
the curvcs of equal heat from west to east, from the basin of the Mis-
sissippi to the eastern coasts of Asia, through an extent of 4000
leagues, we are struck with the great regularity which appears in the
variations of the winter temperature.
Baron Humboldt on Isothermal Lines. 301
J* Differences of the Seasons from the Equator to the Polar Circle.
Isnihtr-
mal
Lines of
68?
59
50
41
Cisatlantic Region.
Long. 1? VV. and 17? E.
Mean Temperature.
Winter. Summer.! Diff.
59-0
446
55 "6
24-8
14-0
?0-6
73-4
68-0
608
53'6
21 6
28"8
32-4
36-0
39-6
Transatlantic Region.
Long. 58??72? W.
Mean Temperature.
Winter, i Summer. Diff.
53 *6
39-2
30-2
14*0
1*4
80-6
78-8
71-6
66-2
55-4
27-0
39*6
41-4
52-2
54-0
This table shows the increase of the difference between the winters
and summers from 28? and 30? to the parallels of 55? and 65?. Thei
increase is more rapid in the Transatlantic zone, where the isothermal
lines of 329 and 50? approach one another very much; but it is re-
markable that, in the two zones which form the two systems of dif-
ferent climates, the division of the annual temperature between winter
and summer is made in such a manner that, upon the isothermal line
of 32?, the difference of the two seasons is almost double of that which
5js observed on the isothermal line of 68?.
Cisatlantic Region.
Long. 31? E. and 22? W.
Places.
( Pondicherry)
Cairo,
Fnnchal,
Rome,
Bourdeaux,
Paris,
Copenhagen,
Stockholm,
Drontheim,
Umeo,
Latitude.
11*55
30-02
32-37
41-53
44*50
48*50
55-41
59-20
63-24
63-50
Mean Temperature.
Whole Year. Winter. Summer,
85 '3
72*7
68-7
60-4
56*5
51*4
45*7
42-3
39-9
33-3
77-0
57-7
63*9
45-9
42-1
38*3
30*7
25-5
24-7
12'9
Transatlantic Region.
Long. 69? E. and 99? W.
Places.
Cumana,
Havannah,
Natchez,
Cincinnati,
lJiiiladelphia,
New York,
Cambridge^
Quebec,
Nain,
Fort. Churchill,
Latitude.
10 '27
23-10
31-28
39-06
39*56
40-40
42*25
46-47
57*10
59-02
Mean Temperature.
WholeYear. Winter. Summer
81*9
78*1
64-8
53-6
54-9
53-8
50-4
41*9
26-4
25*3
81*7
71*2
48-6
32-9
32-2
29-8
34*0
14*2
0*6
6-8
502 Collectanea Medica.
If, instead of the mean temperatures of the seasons, we consider, I
do not say the days of the maxima and minima of the year, whieh arc
the ordinates of the concave and convex summits of the entire curve,
but the mean temperatures of the warmest and the coldest month, the
increase of the differences becomes still more perceptible. We re-
quest the reader to compare, in the following table, only the places
which belong to regions bounded by the same meridians, and conse-
quently to the same system of climate; as, for example, to the region
of Eastern America to that of Western Europe and that of Eastern
Asia. Wc must also attend to the changes of temperature produced
by the monsoons in a part of the equinoctial regions, and distinguish,
under the temperature zone, between the climate of the interior, or
the continental climate, and that of islands and coasts.
Places.
Cuniana,
Pondicherry,
Manilla,
Vera Cruz,
CapeFrangais
Havannah,
Funchal,
Natchez,
Cincinnati,
Pekin,
Philadelphia,
New York,
Rome,
Milan,
Buda,
Paris,
Quebec,
Lilt.
10*27
11 "55
14*56
19*11
19*46
23*10
32*37
31*28
39* 6
of)'5 4
39*56
40-40
41*53
45-23
47*29
48-50
46*47
53*21
55*68
52-14
$9-56
71- 0
Mean Tanperature.
Coldest Wannest
Month, Month.
80-1
761
68-0
70-0
77-0
70-0
64-0
46-9
29-6
24-8
29-8
25-3
42*1
33*8
27 -7
35-1
14-0
37*6
3&'?
27-1
8-r,
22*1
84-4
91*4
86-9
81*7
86-0
83-8
75*6
78*8
74*4
84-2
770
80-8
77-0
55-2
71-6
69-8
73-4
60-3
59-4
p70-3
"65*7
46-6
Differ-
ence.
4*3
15*3
18-9
11*7
90
13-8
11*6
31*9
44*8
59*4
47*2
55'5
34-9
21*4
43-9
34-7
59*4
211
43*2
57*1
24-5
Observations.
Uninterrupted trade-winds.
Monsoons. Radiat. oF the sands.
Monsoons.
North winds in winter.
Uninterrupted trade-winds.
North winds in winter.
Insular climate.
Transatlantic region. Interior.
Same system of climate.
Region of Eastern Asia.
Transatl. region. Eastern coasts.
Idem.
Cisatlantic region. '
Inter or la d.
Idem.
Nearer the western coast.
Transatl. region. Eastern coasts.
Region of the West of Europe.
Insular climate.
Idem.
Interior land.
East of Europe.
Climate of coasts and islands.
We may conclude, in general, that, for any given place in the curves
which express the annual temperatures, the ordinates of the concave
and convex summits difl'er the more from one another, as the tempera-
tures diminish. In the New World, under 40? of latitude, we find a
greater difference between the warmest and coldest months of the year
than in the Old World, at Copenhagen and Stockholm, under 56??
59? of latitude. At Philadelphia, the thermometer descends to 50? or
59? below the freezing-point, while, under the same parallel in Europe,
it descends scarcely 30*6 below it.
On high mountains in islands of little extent, and along the shores,
the lines of annual temperature take nearly the same form as in warm
climates, having only a less degree of curvature. The difference be-
tween the seasons, too, becomes smaller. At the North Cape, in 71?
Baron Humboldt on Isothermal Lines. 303
of latitude, and in the isothermal line of 32?, it is almost 11? greater
than at Paris, in 49? of latitude, and in the isothermal line of 50?.
The sea-breezes and the fogs which render the winters so temperate,
diminish at the same time the heats of summer.* The characteristic of
any climate is not the difference between the winters, expressed in
degrees of the thermometer: it is this difference, compared with the
absolute quantities indicated by the mean temperature of the seasons.
II. Difference between the Winters and Summers, in following the
same Isothermal Line from West to East.
The differences between the seasons of the year are less great near
the convex summits of the isothermal curves, where these curves rise
again towards the North Pole, than near the concave summits. The
same causes which affect the inflexion or the greatest curvature of the
isothermal lines, tend also to equalize the temperatures of the seasons.
The whole of Europe, compared with the eastern parts of America
and Asia, has an insular climate; and, upon the same isothermal line,
the summers become warmer anil the winters colder, in proportion as
we advance from the meridian of Mont Blanc towards the east or the
west. Europe may be considered as the western prolongation of the
old continent; and the western parts of all continents are not only
warmer at equal latitudes than the eastern parts, but, even in the zones
of equal annual temperature, the winters are more rigorous and the
summers hotter on the eastern coasts than upon the western coasts of
the two continents.
The mean temperature of the year being equal to the fourth part of
the winter, spring, summer, and autumnal temperatures, we shall have
upon the same isothermal line of 53"6 (12? cent.)
At the concave summit in America, ) -3.^; ? 32? -fr* 52*3 + 75 6 + 54 j
74*40 West long. $ 4
At the convex summit in Europe, j,?, 40'1 +.51'8 + 68*4 + a4'l
2*20 West long. \ J 4
At the concavc summit in Asia, )   ?24*8+54'7 + 80-6 +54*3
116*20 East long.
n Asia, ) ? ? 24't
' > 53*6 =
This analogy between the eastern coaits of Asia and America suffi-
ciently proves that the inequalities of the seasons, of which we have
endeavoured to fix the numerical relations, depend on the prolongation
and enlargement of continents towards the pole; of the size of seas in
relation to their coasts; and on the frequency of the N.W. winds,
which are thevent* de Remous of the temperate zone; and not on the
proximity of some plateau, or elevation of the adjacent lands. The
great plateaus of Asia do not stretch beyond 52? of latitude; and, in
the interior of the New Continent, all the immense basin bounded by
the Alleghany range and the rocky mountains, and covered with secon-
dary formations, is not more than from 656 to t)20 feet above tho level
of the ocean, according to the levels taken in Kentucky, on the banks
of the Monongahela, at Lake Erie.t
* Leopold von Buch's Travels in Lapland, torn- ii.
t Drake's Nat. and Statist, View of Cincinnati, p-
304 Collcctanca Medica.
The following table indicates, for all the habitable temperate zone;
the division of the same quantity of annnal heat between the two sea-
sons of winter and summer. The numbers which it contains are cither
the result of direct observations, or of interpolations between a great
number of observations made in neighbouring places and situated un-
der the same meridian. We have followed each isothermal curve from
?west to east, giving the preference to places situated near the summits
of the curve, as presenting at the same time the greatest differences in
the distribution of the annual heat. The longitudes are techoned
from the observatory of Greenwich.
Isothermal Lines from 32? to 6'8C
Isoth. Line*
of 68?. i
Isoth. Line J
of 63-5. i
Isoth. Line \
of 59?. (
Isoth. Line^
of 54*5.
Isoth. Line,
of 50?.
Isoth.
of 45 5.
Isoth.
of
Isoth.
of 36":
Isoth. Line^
of 32?. i
Long.
82*10 W.
16-56 W.
3' 0 E.
89-40 W.
14-11 E.
84-10 W.
3??4?E.
84-40 W.
74*10 W.
1-32 W.
9-20 E.
116-20 E.
84-20 W.
71-10 W.
6-40 W.
0-40 W.
2-20 E.
19- 0 E.
116-20 E,
71- 0 W.
2-10 W.
12-35 E.
21-20 E.
71-10 W.
9-20 E.
17-20 E.
24-20 E.
36-20 E.
71-40 W.
18- 5 E.
22-20 E.
57-40 E.
19-50 E.
25-20 E.
Lat.
29*30
32-37
36-48
32-30
40-50
35'30
43-30
38-30
40- 0
47-10
45-30
40- 0
41*20
42-30
52-30
53"30
51* 0
47-30
40- 0
44-42
57- 0
55-40
53- 5
47- 0
62-45
60-30
60- 0
58-30
50- 0
62-30
62-50
53- 0
65- 0
71* 0
Mean Temperature.
Winter. Summer,
Florida,
Madeira,
North Africaj
Mississippi,
Italy,
Basin of the Ohio,
Middle of France,
America, West of Alleghany,
America, East of ditto,
West of Fiance,
Lombardy,
Fast of Asia,
America, West of Alleghany,
America, East of ditto,
Ireland,
England,
Belgium,
Hungary,
Eastern Asia, ?
America, East of Alleghany,
Scotland,
Denmark,
Poland,
Canada,
West of Norway,
Sweden,
Finland,
Central Russia,
Canada,
5 West coast of Gulf of
I Bothnia,
East coast of ditto,
Labrador,.
Sweden,
North Extremity of Norway,
53-6
63-5
59-0
46*4
500
39-2
44-6
34'7
32-5
39-0
34-7
26 6
31-1
30-2
39-2
37-4
36-5
31-1
23-0
23 9
36*1
30-3
28-0
14-0
24-8
24-8
23-0
22-1
6-8
17-6
16-5
3-2
11-5
23-9
The following table shows the oscillations, or the maxima and mi-
nima, observed in the division of the heat between the seasons. 1
have added the means of the winters and summers found at different
degrees of longitude, and undc r the same isothermal line.
3
Baron Humboldt on Isothermal Lines. S03
sf
41
50
59
68
Degrees
vf Long,
examined.
63
107
200
87
84
Oscillations observed in the
Means.
Wilder s. \ Sumners.
3*2 to 24-8 51 8 to 53'6
14'0 24-8 6i-6 68*0
23 0 37-4 62-6 78*8
39 0 44-6 75*2 77' 0
53-6 59'0 71*6 806
i ?
Means calculated.
Winters. Summers.
14-0
19*4
30-2
41-9
56'3
The deviations round the mean,?that is, the inequality of the win-
ters on the same isothermal line,? increase in proportion as the annual
heat diminishes, from Algiers to Holland, and from Florida to Penn-
sylvania. The winters of the curve of 68? are not found upon that of
51?, and the winters of 51? are not met with on the curve of 42?. In
considering separately what may be called the same system of climate,
?for example, the European region, the Transatlantic region, or that
of Eastern Asia,?the limits of the variations become still more nar-
row. Wherever, in Europe, in 40? of longitude, the njean tempera-
ture rises <
To 59-0 r 44-6 to 46*4
50-0 C The fntcrs ) 31-1 3?'4 ^al,d th% sum^
45-5 ( aref,'?m > 28-4 36" < mCr5from
41*0) ( 20-3 26
a
;)
In tracing jive isothermal lines between the parallels of Rome and
Petersburg, the coldest winter presented by one of these lines is not
found again on the preceding line. In this part of; the globe, those
places whose annual temperature is 54*5, have not a winter below 32?,
which is already felt upon the isothermal line of 50?. If, in place of
stopping at the most rigorous winter which each curve presents, wc
trace the lines of equal winter temperature, (or the isocheimal lines,)
these lines, instead of coinciding with the lines of equal annual heat?
oscillate round them. As the isocheimal lines unite points placed on
different isothermal lines, we may examine to what distance their
summits extend. In considering always the same system of climates,
?for example, the European region,?we shall find that tjic lines of
equal winter cut isothermal lines which are nine degrees distant. In
Belgium, (in latitude 52?, and in isothermal latitude 51'8,) and even
in Scotland, (in latitude 57?, and isothermal latitude 45'5,) the win-
ters are more mild than at Milan, (in latitude 45*28,) and in isother-
mal latitude 55*8,) and in a great part of Lombardy. Farther to the
north, in the Scandinavian peninsula, we meet with three very different
systems of climate:?viz. 1, the region of the west coasts of Norway
to the west of the mountains; 2, the region of the eastern coasts of
Sweden to the east of the mountains ; and, 3, the region of the west
coasts of Finland, along the gulf of Bothnia. No where, without the
tropics, is the division of the annual heat among the seasons more
equal. In the temperate zone, under parallels nearer to our own,
Ireland presents an example still more striking of the union of very
wild winters with cold and moist summers. Notwithstanding a difier-
no. 271. 2 R
306 Collectanea Medica.
ence of four degrees of latitude, the ^inters there arc as mild as in
Britain, while the mean temperature of the summers is three degrees
less. This is the true maritime climate. The month of August, which
on the same isothermal line, in the east bf Europe,* in Hungary, has
the temperature of 71*6, reaches only 60-8 at Dublin. The month of
January,-whose mean temperature at Milan, and in a great part of
Lombardy, is only 35 6, rises in Ireland to 5*4 and 7*2. On the
coasts of Glenarm, also, (in north lat. 54*56,) under the parallel of
Konigsberg, the myrtle vegetates with the same strength as in Portugal.f
It scarcely freezes there in winter, but the heat of summer is not ca-
pable of ripening the vine.
These examples are sufficient to prove that the isocheimal lines
deviate much more than the isothermal lines from the terrestrial paral-
lels. In the system of European climates, the latitudes of two places
that have the same annual temperature cannot differ more than from
four to five degrees, while two places whose mean winter temperature
is the same may differ more than nine or ten degrees in latitude. The
farther we advance to the east, the more rapidly do these differences
increase.
The lines of equal summer, or isolheral curves, follow a direction
exactly contrary to the isocheimal lines. We find the same summer
temperature at Moscow, in the centre of Russia, aud towards the
mouth of the Loire, notwithstanding a difference of eleven degrees of
latitude. Such is the effect of the radiation of the earth on a vast con-
tinent deprived of mountains. It is sufficiently remarkable that the
inflexions of the isothermal lines, and the division of lands and seas,
are such upon the globe that every where, in North America, in
Europe, and in Eastern Asia, the mean temperature of the summers
does not denote more than 36? in the parallels of from 45? to 47??
The same causes which, in Canada and in the north of China, sink the
curves of equaj annual heat, where the isothermal lines (those of 51*8
and 53'6,) corresponding to the parallels of 45? and 47?, tend to
raise the lines of equal summer, or the isotheral curves.
* Walilenberg Flora Carpath. p. 90.
t Irish Transactions, torn. viii. p. 116, 203, 269.

				

